# Supplementary material for: Multifunctional Oxidized Succinoglycan/Poly(N-isopropylacrylamide-co-acrylamide) Hydrogels for Drug Delivery
Source: Polymers (Basel). 2022 Dec 28;15(1):122. doi: 10.3390/polym15010122 (PMC9824477; doi:10.3390/polym15010122)
Supplement: Supplementary file 1 [file polymers-15-00122-s001.zip › polymers-2096679-supplementary.pdf]

Supporting Information

Multifunctional oxidized succinoglycan/poly(N-isopropylacrylamide-co-acrylamide) hydrogels for drug delivery

Yiluo Hu [1], Younghyun Shin [1], Sohyun Park [1], Jae-pil Jeong [1], Yohan Kim [1] and Seunho Jung [1,2,*]

[1] *Department of Bioscience and Biotechnology, Microbial Carbohydrate Resource Bank (MCRB), Konkuk University, Seoul 05029, Korea*

[2] *Department of Systems Biotechnology, Microbial Carbohydrate Resource Bank (MCRB), Konkuk University, Seoul 05029, Korea*

** Correspondence: shjung@konkuk.ac.kr; Tel.: +82-2-450-3520*

Table of content

1. Chemical structure analysis of OSG (**Figures S1 and S2**)
2. Thermal analysis of hydrogels. (**Figures S3 and S4**).
3. Morphological analysis of OSG/P(NIPAM-AM) hydrogels. (**Figures S5 and S6**)
4. Dug loading amount of hydrogels. (**Table S1**)

5. Hydrogel degradation test (**Figure. S7**)

1. The chemical structure analysis of OSG

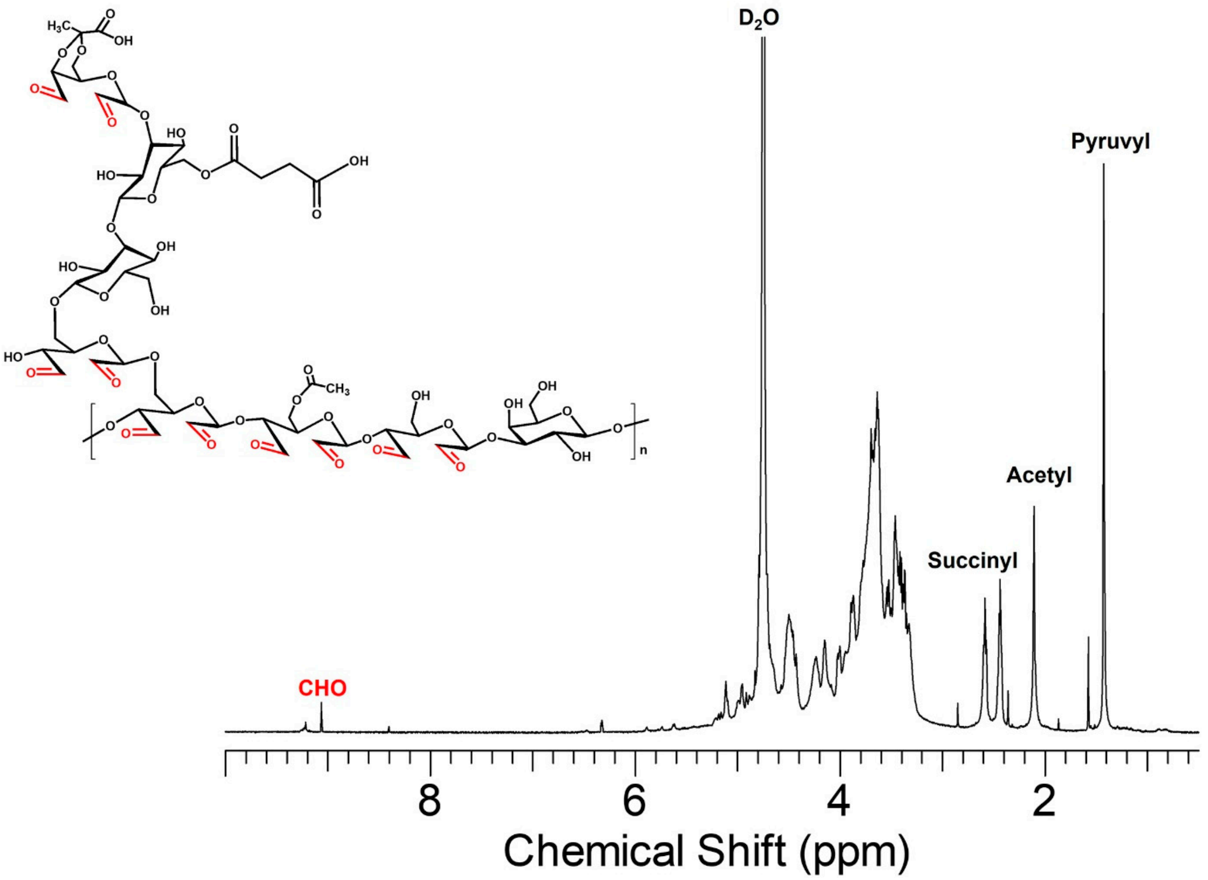

Figure S1. ^{1}H NMR spectrum of OSG. NMR spectra were recorded with a 500 MHz Bruker Avance (Bruker, Karlsruhe, Germany) spectrometer. The samples were dissolved in deuterated water (D2O, 99.96%), and NMR measurements were performed in a 5 mm NMR tube, at 25 °C.

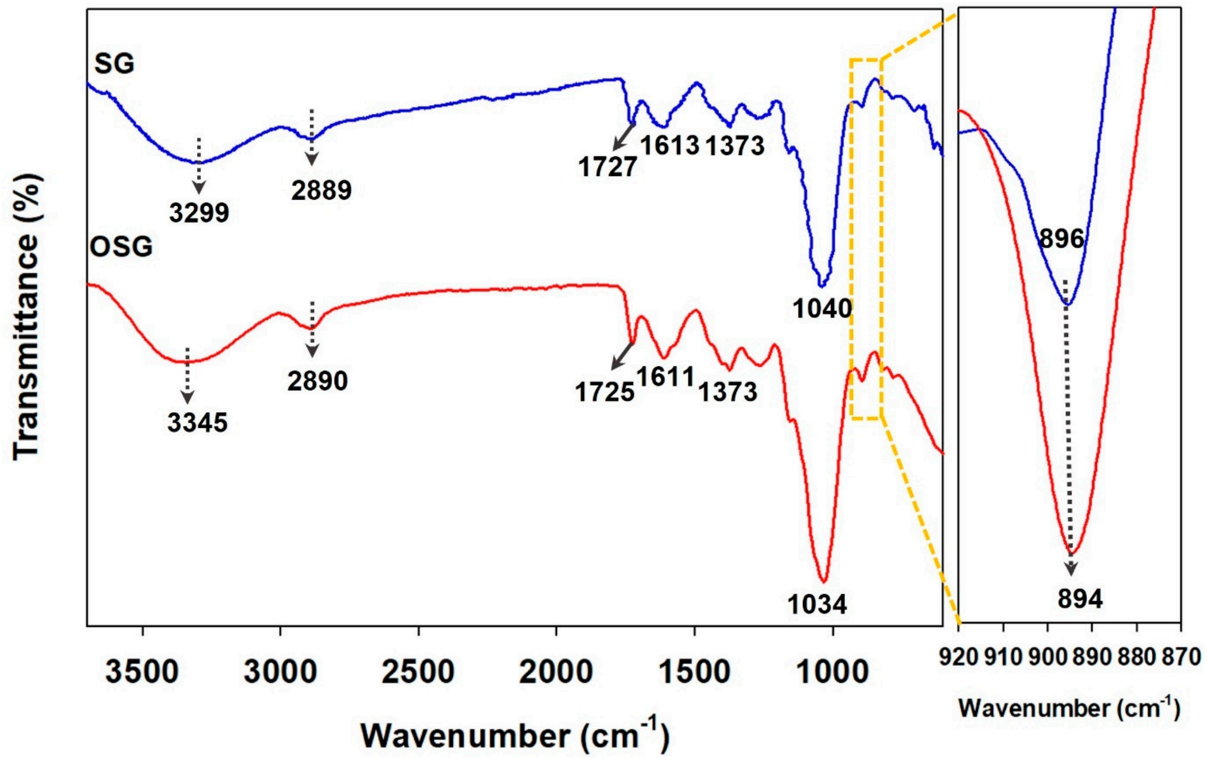

Figure S2. ATR-FTIR spectra of SG and OSG.

2. Thermal analysis of hydrogels.

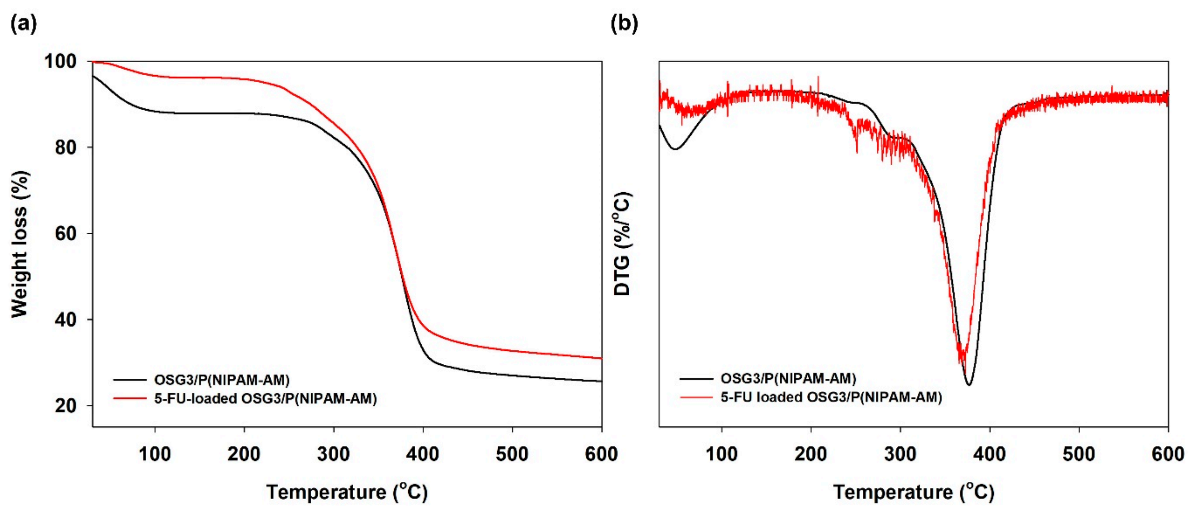

Figure S3. TGA and DTG curves of 5-FU loaded OSG3/P(NIPAM-AM) hydrogels. After 5-FU loading, a similar degradation pattern was observed when compared to OSG/P (NIPAM-AM), but a 69% mass loss was observed in the secondary degradation region. As the 5-FU molecule was loaded onto the gel, it exhibited high thermal stability and was capable of heat-induced release, suggesting that it could be applied as an anti-cancer drug delivery system [1].

[1] Reddy, P.R.S.; Rao, K.M.; Rao, K.S.V.K.; Shchipunov, Y.; Ha, C.S. Synthesis of Alginate Based Silver Nanocomposite Hydrogels for Biomedical Applications. Macromol. Res. 2014, 22, 832–842

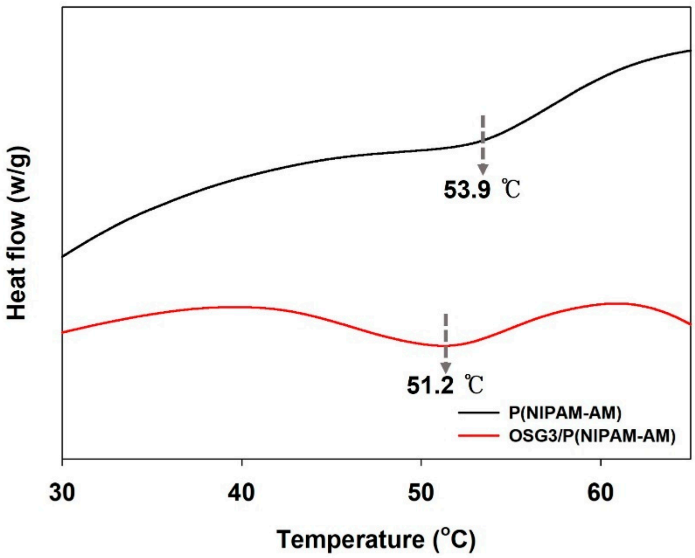

Figure S4. DSC curves of P(NIPAM-AM) and OSG3/P(NIPAM-AM). Differential scanning calorimetry (DSC) was conducted using SDT Q600 thermal analyzer Q 20 (TA Instruments, USA) from 25 °C to 80 °C with a scanning rate of 10 °C/min. The P(NIPAM-AM) gel showed an endothermic peak at 53.9 °C in the DSC curve. When OSG was introduced into the P(NIPAM-AM) hydrogel, an endothermic peak appeared at a lower temperature (51.2 °C) than in the control group.

3. Morphological analysis of OSG/P(NIPAM-AM) hydrogels.

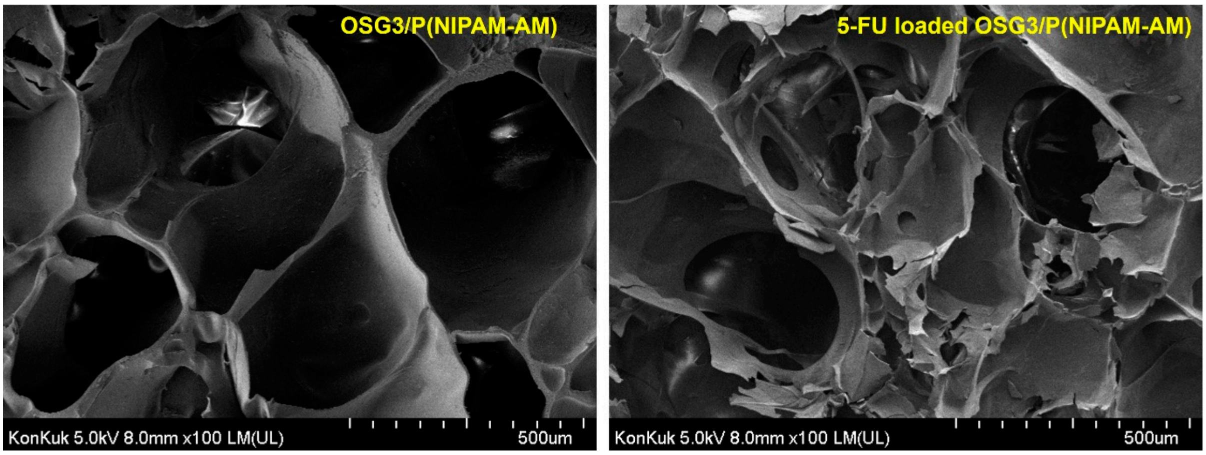

Figure S5. FE-SEM images of OSG3/P(NIPAM-AM) hydrogels (left) and 5-FU loaded OSG3/P(NIPAM-AM) (right).

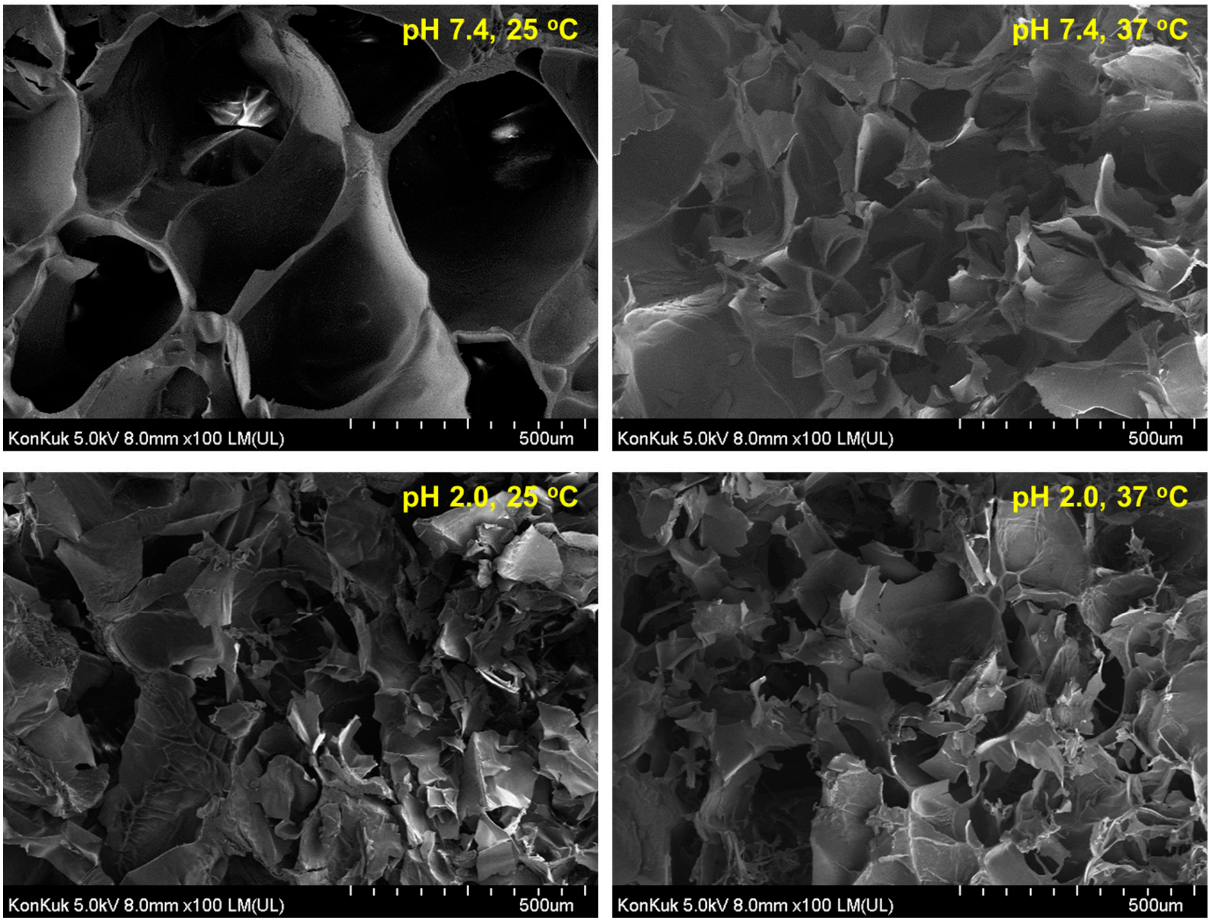

Figure S6. FE-SEM images of OSG3/P(NIPAM-AM) after swelling in different temperatures and pH values. OSG3/P(NIPAM-AM) hydrogels were swelled in solution in pH (7.4 and 2.0)

and temperature (25, and 37 ^{o}C) solutions, respectively. Equilibrium-swollen hydrogels were lyophilized at -85 ^{o}C.

4. Dug loading amount of hydrogels

The 5-FU loading of hydrogels encapsulated the drug in a swelling equilibrium method. Drug loading was performed at room temperature for 48 h by immersing the dried hydrogels in 5 mL of an aqueous drug solution (1 mg/mL) and swelling. After drying the hydrogel loaded with the next drug, it was stirred in 5 mL of PBS solution for 12 h. The absorbance of the supernatant obtained by centrifugation was then recorded at 265 nm using a UV-Vis spectrophotometer. The calibration curve under the concentration range is linear.

$$pH = 7.4, y = 67.36x + 0.0104, R^2 = 0.9999$$

$$pH = 2.0, y = 51.83x + 0.0167, R^2 = 0.9998$$

Based on this formula, the loading result of 5-FU in the hydrogel is shown below.

Table S1. Summarizing the drug loading content of the hydrogels.

**Samples**	**Dry weight (mg)**	**Loading amount (mg/g)**	**Encapsulation Efficiency (%)**
**P(NIPAM-AM)**	207	20	16.6
**OSG1/P(NIPAM-AM)**	214	21	17.4
**OSG2/P(NIPAM-AM)**	209	17	14.5
**OSG3/P(NIPAM-AM)**	179	26	20.1
**OSG4/P(NIPAM-AM)**	163	23	17.4

5. Hydrogel degradation test

In the degradation test, the hydrogel was immersed in pH 7.4 buffer and pH 2.0 buffer, respectively, and cultured with shaking at 100 rpm at a constant temperature of 37 ° C. It was determined by removing moisture from the gel surface at a certain time interval and calculating the mass difference.

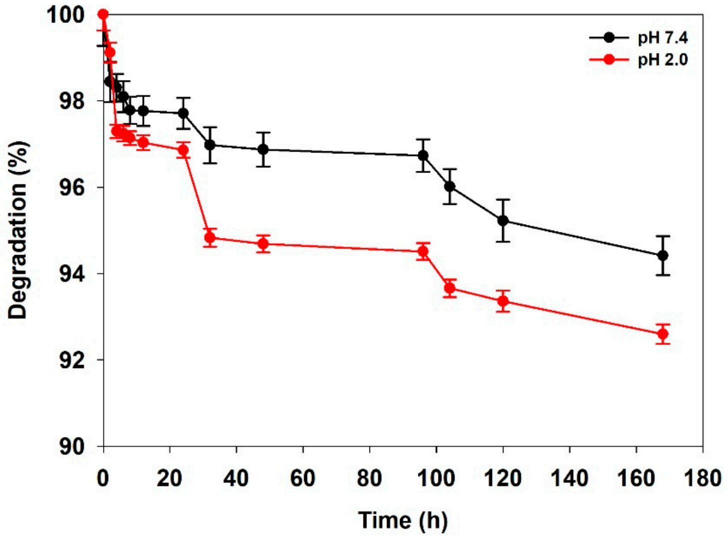

Figure S7. Degradation rates of the hydrogels in pH 7.4 and pH 2.0. The degradation behavior of the hydrogel was monitored by the weight loss method at pH = 7.4 and pH 2.0. The weight of the hydrogel decreased steadily over time, and a difference in degradation rate was observed at pH 7.4 and 2.0. This suggested that the Schiff-base formation inside the hydrogel was more easily degraded by acidic pH.
